# [Corrigendum] ACSM3 suppresses proliferation and induces apoptosis and cell cycle arrest in acute myeloid leukemia cells via the regulation of IGF2BP2

**DOI:** 10.3892/etm.2024.12663

**Published:** 2024-07-25

**Authors:** Xin Zheng, Jinjun Wu, Linlan Song, Bo Huang

Exp Ther Med 25:177, 2023; DOI: 10.3892/etm.2023.11876

Subsequently to the publication of this paper, the authors informed the Editorial Office that, for the western blots shown in Fig. 5H on p. 7, the data shown correctly for the Cyclin D1 experiment had inadvertently been duplicated for the control GAPDH experiment.

The revised version of Fig. 5, now featuring the correct protein blot for the GAPDH control experiment in Fig. 5H, is shown on the next page. Note that this error did not have a major impact on the conclusions reported in this study, and all the authors agree to the publication of this Corrigendum. The authors thank the editor of *Experimental and Therapeutic Medicine* for granting them the opportunity to publish this Corrigendum, and apologize to the readership for any inconvenience caused.

## Figures and Tables

**Figure 5:**
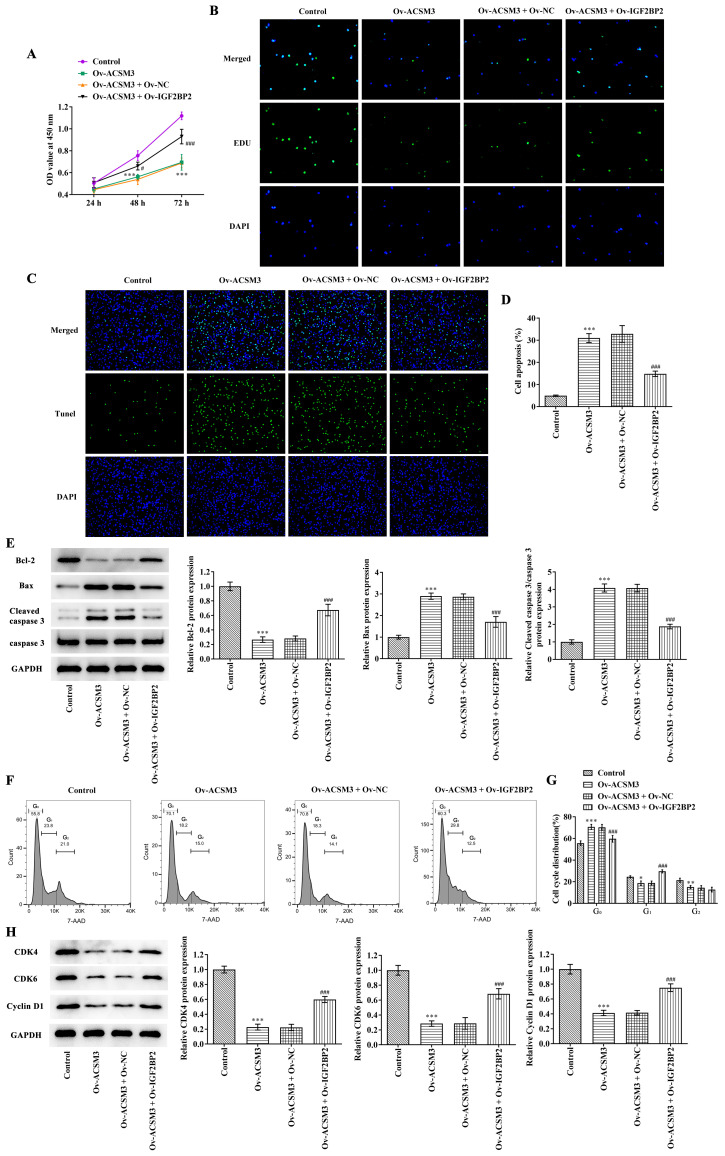
Effects of ACSM3 on the proliferation, apoptosis and cell cycle of HL-60 cells is associated with regulation of IGF2BP2. Cell proliferation was evaluated using (A) CCK-8 assay and (B) EdU staining. (C) Apoptosis was detected and (D) quantified using TUNEL assay. (E) Western blotting was used to assess the protein levels of Bcl-2, Bax and cleaved caspase 3/caspase 3. (F) Cell cycle was detected and (G) quantified using flow cytometry analysis. (H) Western blotting was used to evaluate the protein levels of CDK4, CDK6 and Cyclin D1. Results are displayed as the mean ± SD. ^*^P<0.05, ^**^P<0.01 and ^***^P<0.001 vs. Control. ^#^P<0.05 and ^###^P<0.001 vs. Ov-ACSM3 + Ov-NC. ACSM3, acyl-CoA medium-chain synthetase-3; EdU, 5-ethynyl-2’-deoxyuridine; Ov, overexpressing; NC, negative control; OD, optical density. Bcl-2, B-cell lymphoma-2; Bax, Bcl2-Associated X; CDK4, cyclin dependent kinase 4; CDK6, cyclin dependent kinase 6.

